# Assessing the validity of two-dimensional video analysis for measuring lower limb joint angles during fencing lunge

**DOI:** 10.3389/fspor.2024.1335272

**Published:** 2024-02-14

**Authors:** Kenta Chida, Takayuki Inami, Shota Yamaguchi, Takuya Nishioka, Yasumasa Yoshida, Naohiko Kohtake

**Affiliations:** ^1^ Graduate School of System Design and Management, Keio University, Yokohama, Japan; ^2^ Institute of Physical Education, Keio University, Yokohama, Japan

**Keywords:** hip, knee, ankle, lunge performance, Qualisys, Frame-DIAS, sagittal plane

## Abstract

**Introduction:**

The fencing lunge (lunge), characterized by minimal body rotation, offers a movement well-suited for 2D video analysis. However, to the best of our knowledge, the validity of 2D video analysis for fencing has not been verified. This study aimed to validate 2D video analysis by comparing lower limb joints (hip, knee, and ankle joints) angles during lunge using both 2D video analysis and 3D motion analysis methods.

**Methods:**

Twenty-two male fencers performed lunge trials that were simultaneously recorded using eight motion capture cameras (Qualisys Miqus M1) and two digital video cameras (Sony AX-450 and AX450a).

**Results:**

The 2D video analysis results exhibited an extremely large correlation in knee joint angles of the front and rear legs in the sagittal with those from 3D motion analysis (*r* = 0.93–0.99). However, while a robust correlation was found between the ankle joint angles of the front and rear legs (*r* = 0.82–0.84), a large bias was also observed (−5.23° to −21.31°). Conversely, for the hip joints of the rear leg, a moderate correlation (*r* = 0.31) and a large bias (−10.89°) were identified.

**Conclusions:**

The results of this study will contribute to the development of coaching using 2D video analysis in competition settings because such analysis can be a useful alternative to 3D motion analysis when measuring the knee joint angle of the front leg and rear leg in the sagittal plane. However, for the ankle joint angle, further research on the optimal shooting position and height of the digital video camera is needed, whereas for the hip joint angle, 3D motion analysis is recommended at this time.

## 1 Introduction

The “fencing lunge (lunge)” is the fundamental attack movement in fencing (1) and is pivotal in the attack phase (2). Previous studies have examined various aspects of the lunge, such as joint angle (2–4), joint angular velocity (2, 4), travel distance (5), and peak velocity (2–4). Specifically, studies have shown that the magnitude of the lower limb joint angles, particularly the rear leg's knee joint peak flexion angle and hip joint peak flexion angle, in the lunge's initial phase is linked to increased peak velocity, which reduces the opponent's reaction time to the attacking action and slows the time to defend (2, 3). This indicates that the visualization of lower limb joint angles is important not only from the perspective of performance evaluation but also for developing evidence-based coaching methods for competitions and injury prevention.

Traditionally, three-dimensional (3D) motion analysis systems have served as a method for analyzing motion skills in fencing, including the evaluation of lower limb joint angles (2–4). This method is a standard approach for kinematic assessment of movement during exercise and is known for its high measurement accuracy. It has been used in numerous studies focusing on movement analysis. However, implementing this method in actual competition settings poses challenges, such as the time required to secure personnel to perform specialized analysis, time required for analysis, significant costs of preparing equipment and facilities, and poor portability of the equipment. Therefore, developing a method that can be easily implemented at competition sites to enable movement evaluation with highly reliable measured values is critical to the development of more evidence-based coaching at competition sites.

In clinical practice, two-dimensional (2D) video analysis has gained recognition for its simplicity and increased number of quantitative methods for analyzing sports movements (6–13). In comparison to 3D motion analysis, 2D video analysis is more cost-effective and portable. Recent advancements in development technology, such as higher video quality and frame rates, have further enhanced the utility of data analysis. The 2D video analysis has shown promising potential, indicating good correlation and agreement with 3D motion analysis (6–8, 10–13). Schurr et al. obtained 2D and 3D lower limb joint (hip, knee, and ankle) angle movements from the sagittal plane during a single leg squat and compared the results using Pearson's correlation (7). The results confirmed a moderate-to-strong correlation (*r* = 0.51–0.93), and the Bland-Altman Plot method also showed strong agreement (hip = 2.60°, knee = 0.74°, ankle = 3.12°). Mousavi et al. analyzed lower limb joint (hip, knee, and ankle) angles during running using 2D video analysis from the sagittal plane, compared them with the results of 3D analysis methods, and reported excellent intraclass correlation coefficients (ICC = 0.68 and 0.59, respectively) relationships between the knee and ankle joints (6). However, the accuracy of lower limb joint angles from cameras placed in the frontal plane was low in previous studies that have validated the accuracy (7), and it is also extremely difficult to capture horizontal plane motion (body rotation) (13). In contrast, 2D video analysis is suitable for evaluations made in the sagittal plane relative to the direction of motion. The fencing lunge movement entails minimal body rotation yet has characteristics similar to movements in the sagittal plane relative to the fencing piste, making it easy to reflect the characteristics of 2D video analysis. However, to our knowledge, the applicability and validity of 2D video analysis in the context of fencing, especially for the fencing lunge, remain unverified to date.

Providing coaches and athletes with simple and reliable feedback data using 2D video analysis is expected to effectively improve movement skills and may serve as a useful teaching tool. This study aimed to validate the use of 2D video analysis by comparing lower limb joint angles (hip, knee, and ankle joints) during the fencing lunge using 2D video analysis and 3D motion analysis. Consistent with previous studies on single-leg squats in clinical practice (7), we hypothesized that the angles of the hip and knee joints in the sagittal plane would yield reliable data.

## 2 Materials and methods

### 2.1 Participants

To validate the 2D video analysis in this study, multiple lunge movements had to be performed under the same conditions. Therefore, fencers with at least 3 years of competition experience and capable of consistently performing lunge movements were recruited. The sample size was calculated using G*Power software (G*Power 3.1.9.6; Heinrich-Heine-Universität Düsseldorf, Düsseldorf, Germany). Based on previous studies investigating the correlation between 3D motion analysis and 2D video analysis of lower limb joints (7, 8), the effect sizes were estimated at 0.86 and 0.64, α level at 0.05, and power (1-*β*) at 0.80. At the minimum, 7–16 participants were required. Taking errors into consideration, this study included 22 Japanese high school and university student fencers (age: 19.2 ± 1.8 years, height: 173.3 ± 6.5 cm, weight: 62.4 ± 8.6 kg, fencing experience: 5.2 ± 1.8 years, expressed as mean ± standard deviation). The participants volunteered after being provided with a detailed information sheet explaining the study's purpose and signed an informed consent form. Additionally, the fencers were confirmed to have no musculoskeletal injuries within 6 months prior to the experiment. This study was approved by the Ethics Committee of the Graduate School of System Design and Management, Keio University (approval number: SDM-2023-045).

### 2.2 Experimental setup

**Figure 1:**
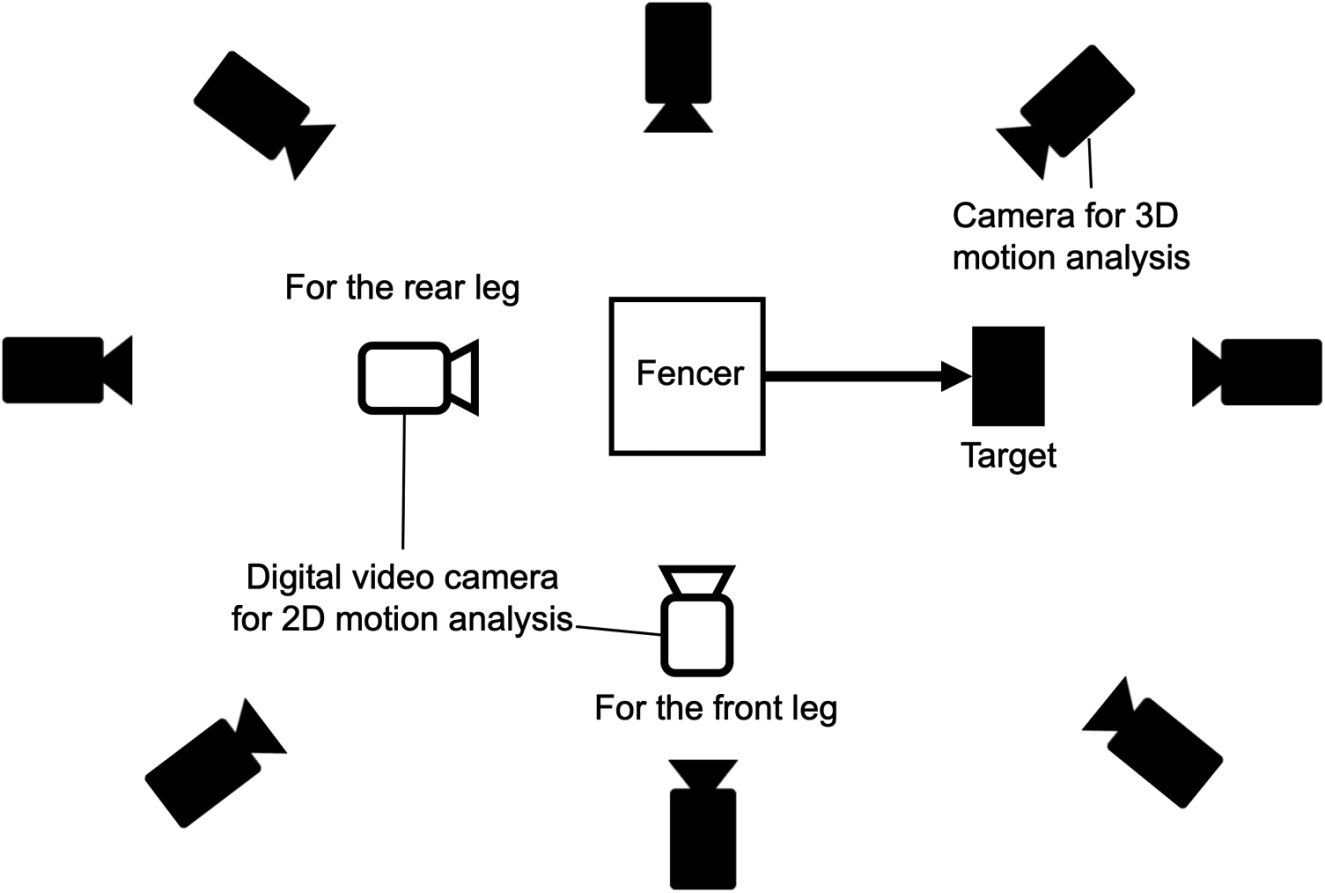
Experimental setup.

Figure 1 shows an experimental setup overview. Lunge distance was defined as the horizontal distance from the toe of the rear leg to the target in the ungirdled state, which was 1.5 × the height of each fencer (3, 14, 15). The target of the attack was a 30 × 30 cm square cloth attached to the target surface, which was positioned to align with the tip of the fencer's lunge-thrusting sword. Prior to the experiment, the participants warmed up for approximately 15 min by stretching, running, and practicing fencing footwork. Familiarization sessions preceded each task to acquaint the participants with experimental conditions and given instructions, including several lunge movements. Starting from a stationary position with both feet grounded, participants performed lunge movements at their discretion (3). Each participant performed three trials for each lunge movement per test session. A trial was deemed unsuccessful if the participant stopped the movement mid-lunge, if the balance of the movement was significantly disturbed, or if the tip of the sword missed the target. A 30 s interval was maintained between test sessions.

### 2.3 Data collection

**Figure 2:**
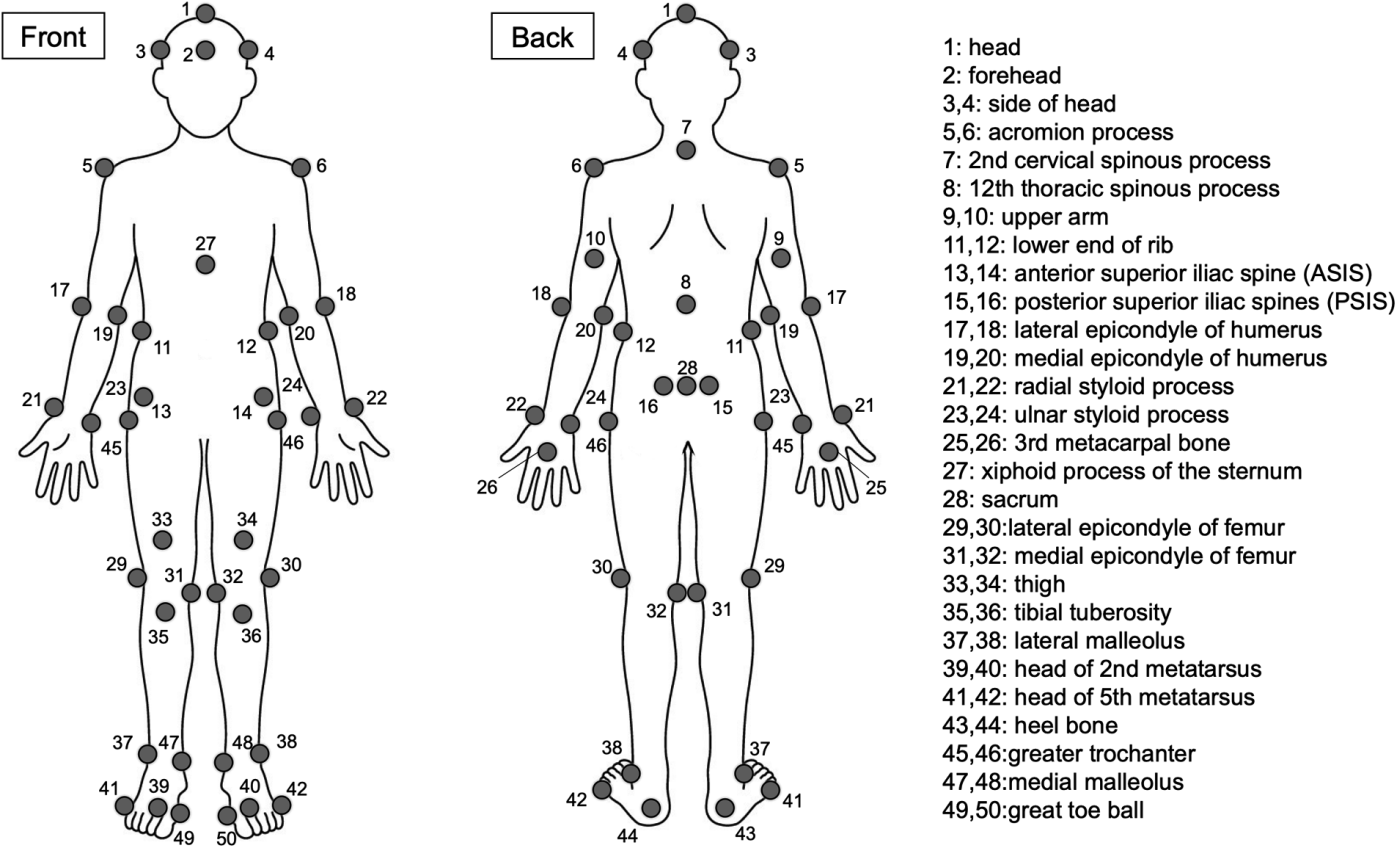
Positions of reflective markers attached to the landmarks of the body.

Reflective markers were attached to the participant's skin using double-sided adhesive tape at 50 points surrounding each joint of the upper and lower limbs, according to the visual 3D Marker Set Guidelines (Figure 2). To reduce measurement errors owing to inconsistencies in marker placement, the markers were placed by one researcher with over 10 years of experience, and the marker positions were further confirmed by another researcher with over 10 years of experience. Participants wore standard equipment, including a fencing mask on their heads, and held a fleuret sword in their dominant hand. A fleuret No. 5 sword (BF Allstar, Germany; blade length: 90 cm) and a mask (Allstar, Germany) conformed to international standards. The participants used their standard fencing shoes.

During the test sessions, 2D and 3D data were collected simultaneously. The 3D data were sampled at 200 Hz using the Qualisys Track Manager 2023.2 (QTM, Qualisys, Goteborg, Sweden). Eight motion capture cameras (Qualisys, Miqus M1, Goteborg, Sweden) were used to collect and record data. A static coordinate system was set up with the y-axis as the direction of lunge motion, the x-axis as the direction orthogonal to the y-axis, and the z-axis as the vertical direction.

Two digital video cameras (AX-450, AX450a, Sony) were used for 2D video analysis. One digital video camera was positioned 3 m behind the participant and the other 3 m to the side of the participant. Thus, for the front leg, the X- and Y-axes were set in the direction of attacking movement, and for the rear leg, the X- and Y-axes were set in the direction perpendicular to the direction of the lunge and in the vertical direction, respectively. In both cases, the participants’ lower limbs were photographed vertically and adjusted to a height of 80.5–88 cm based on the height of the greater trochanter during the “En Garde” of each participant (9), 2D data were collected at 120 frames per second (fps).

### 2.4 Data analysis

The evaluation variables included joint angles of the hip, knee, and ankle joints in the front and rear legs, observed from both the 3D and 2D sagittal planes. The two assessment time points were identified as heel-off and heel-strike, with joint angle data analyzed at each time point. These time points referred to the commencement (heel-off) and termination (heel-strike) of the lunge (1) and were defined as follows:

Heel-off: For the 2D analysis, the initial visible point where the player's front foot heel clearly leaves the ground from the “En Garde” (initial position) position. For the 3D analysis, the height of the z-axis of the heel marker at the En Garde was defined as the minimum height of the heel marker, and the first time it became positive, it was defined as heel-off.

Heel-strike: For the 2D analysis, the initial point where the player's front foot heel makes clear contact with the ground. In 3D analysis, this time point was defined as the time when the z-axis height of the front foot heel marker was first minimized.

The coordinate data derived from the 3D motion analysis using the QTM were processed and labeled. The labeled kinematic data were then exported to Visual3D software (Version 6.0, C-Motion, Inc., Germantown, MD, USA) to calculate the lower limb joint angles. The cutoff frequency was selected based on the residual analysis method (3, 16, 17), and smoothing was performed with an 8 Hz fourth-order Butterworth low-pass digital filter. All segments (head, thorax, upper arm, forearm, hand, pelvis, thigh, shank, and foot) were constructed, and a local coordinate system was established for each segment. Pelvic segments were derived from ASIS and PSIS marker positions, and hip joint centers were calculated using Visual 3D regression equations derived from Bell et al. (18). The knee joint center was defined as the midpoint between the lateral epicondyle of the femur and medial epicondyle of the femur. The ankle joint center was defined as the midpoint between the lateral malleolus and medial malleolus. Joint angles at the hip, knee, and ankle joints were then defined using these segments (Table 1).

**Table 1:** Definition of measured variables.

Variables	2D Definitions	3D Definitions
Sagittal plane hip angle	The offset angle between the line drawn from the acromion marker to the greater trochanter marker and that drawn from the greater trochanter marker to the lateral femoral epicondyle marker (7, 9).	The angle between the pelvis and thigh in the sagittal plane.
Sagittal plane knee angle	The offset angle between the line drawn from the greater trochanter marker to the lateral femoral marker and that drawn from the lateral femoral marker to the lateral malleolus marker. (7, 9).	The angle between the thigh and shank in the sagittal plane.
Sagittal plane ankle angle	The offset angle between the line connecting the lateral femoral epicondyle marker and the lateral malleolus marker and the line connecting the heel marker and the fifth metatarsal marker (10).	The angle between the shank and foot in the sagittal plane.

**Figure 3:**
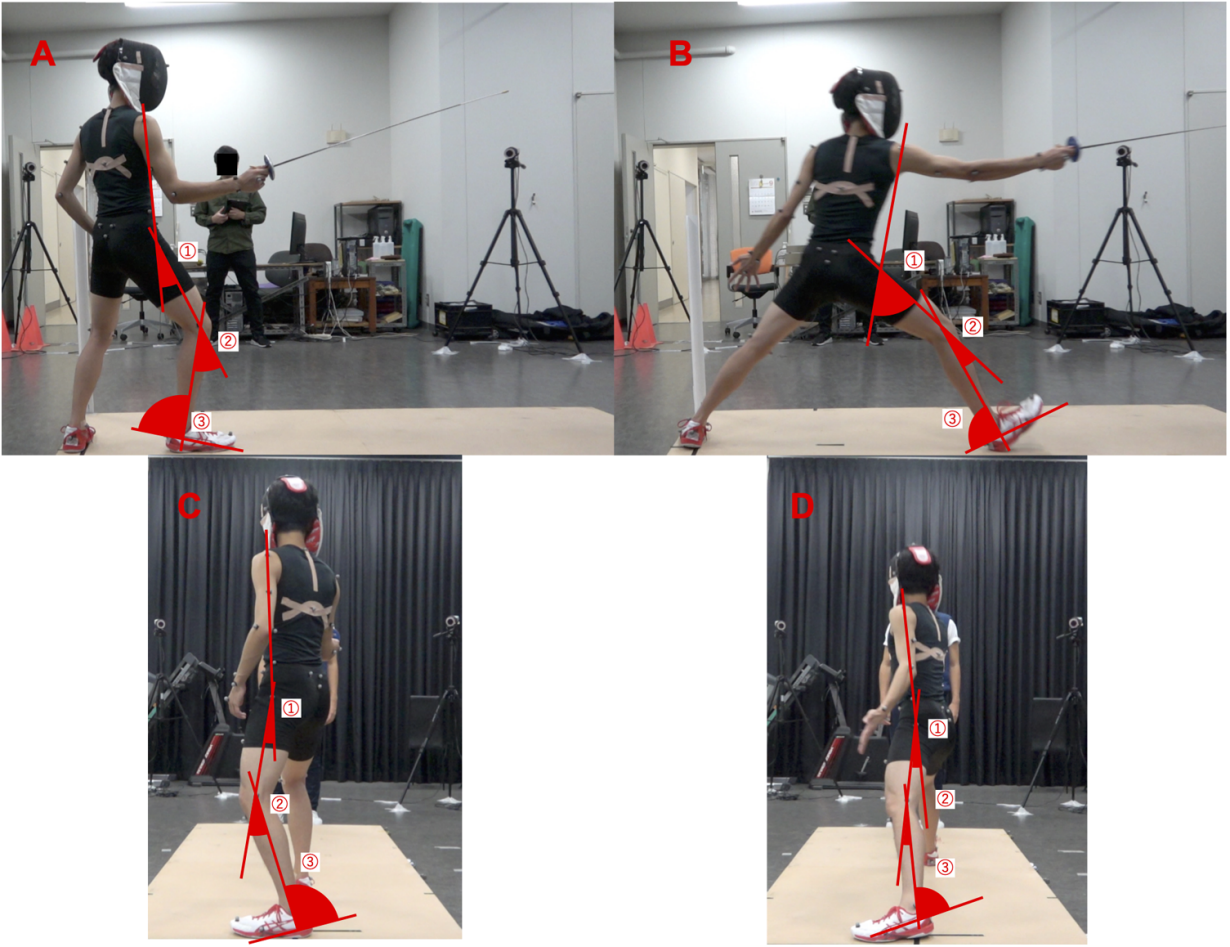
Screening information for sagittal hip, knee, and ankle angles during heel-off and heel-strike of front and rear legs. (**A**) Sagittal plane front leg at heel-off; (**B**) sagittal plane front leg at heel strike; (**C**) sagittal plane rear leg at heel-off; and (**D**) sagittal plane rear leg at heel strike; (1) hip joint angle; (2) knee joint angle; and (3) ankle joint angle.

All 2D motion analysis methods were performed using Frame-DIAS 6 (DKH Inc, Tokyo, Japan), a reliable tool that has been validated in several biomechanical studies (19–21). For the hip joint angle, the offset angle was set between the line drawn from the acromion marker to the greater trochanter marker and that drawn from the greater trochanter marker to the lateral femoral epicondyle marker (7, 9). For the knee joint, the offset angle was set between the line drawn from the greater trochanter marker to the lateral femoral marker and the line drawn from the lateral femoral marker to the lateral malleolus marker (7, 9). For the ankle joint, the offset angle was set between the line connecting the lateral femoral epicondyle marker to the lateral malleolus marker and the line connecting the heel marker to the fifth metatarsal marker (10) (Table 1 and Figure 3). In a preliminary pre-study experiment, Frame-DIAS 6 (2D video analysis) reliability test was conducted based on resting position before the start in 10 randomly selected participants. Analysis of two joint angle variables at the hip, knee, and ankle joints in the fore and hind legs showed strong reproducibility in intra-rater reliability tests {mean absolute difference = 0.10°±0.64°, mean ICC 1,1 = 0.985 [95% confidence interval (CI) 0.945–0.996]}. All 3D and 2D data were evaluated by analyzing the means of three trials (7).

### 2.5 Statistical analysis

Data were tested for normality using the Shapiro–Wilk test and analyzed using Pearson's correlation coefficient and Bland–Altman plots to assess the relationship and agreement between 2D video cameras and 3D motion capture (22, 23). The strength of Pearson's correlation (r) was defined as <0.3 (small), 0.3–0.49 (moderate), 0.5–0.69 (large), 0.7–0.89 (very large), and 9.0–1.0 (extremely large) (24). The significance level was set at 5%. Statistical analyses were performed using IBM SPSS Statistics (Version 29.0.1.0, IBM Corp., Armonk, NY., USA) for single regression analysis, and Pearson's correlation coefficient and Bland-Altman plots were generated using Microsoft Excel software (Version 14.4.0, Microsoft Corporation, Redmond WA, USA).

## 3 Results

The final set distance between the fencer and the target was 260.0 ± 9.8 cm. Comparative data for each joint angle variable using 2D and 3D images are presented in Table 2.

**Table 2:** Comparison of each outcome of joint kinematics assessment between 3D and 2D data collection methods.

			3D means	2D means	R	*p*	95%CI	Mean difference 3D-2D	95% limits of agreement (lower-upper)
Front	Hip	Heel off (°)	40.25 ± 10.05	33.97 ± 9.80	0.71	<.001	.419–.873	6.28 ± 7.51	−8.44–20.99
		Heel strike (°)	62.28 ± 13.76	57.34 ± 8.77	0.90	<.001	.774–.959	4.93 ± 6.98	−8.74–18.61
	Knee	Heel off (°)	48.47 ± 10.09	44.16 ± 8.85	0.96	<.001	.905–.984	4.31 ± 2.95	−1.46 to −10.08
		Heel strike (°)	23.70 ± 5.39	23.71 ± 5.90	0.93	<.001	.829–.970	0.001 ± 2.22	−4.35 to −4.34
	Ankle	Heel off (°)	71.58 ± 5.33	77.93 ± 5.01	0.83	<.001	.629–.927	−6.35 ± 3.03	−12.29 to −0.42
		Heel strike (°)	64.56 ± 6.83	85.86 ± 6.70	0.83	<.001	.634–.928	−21.31 ± 3.91	−28.98 to −13.64
Rear	Hip	Heel off (°)	11.90 ± 9.30	16.22 ± 8.33	0.81	<.001	.588–.918	−4.33 ± 5.52	−15.15–6.50
		Heel strike (°)	−3.63 ± 5.95	7.25 ± 6.27	0.31	.163	−.130–.646	−10.89 ± 7.19	−24.98–3.20
	Knee	Heel off (°)	40.19 ± 11.23	36.64 ± 10.81	0.99	<.001	.986–.998	3.55 ± 1.27	1.07–6.03
		Heel strike (°)	13.72 ± 6.89	14.89 ± 7.10	0.98	<.001	.958–.993	−1.17 ± 1.32	−3.76–1.42
	Ankle	Heel off (°)	86.53 ± 5.74	91.76 ± 6.10	0.82	<.001	.610–.923	−5.23 ± 3.57	−12.22 to 1.76
		Heel strike (°)	66.37 ± 8.24	75.35 ± 7.35	0.84	<.001	.658–.934	−8.98 ± 4.42	−17.64 to −0.31

Data are presented as mean ± standard deviation. hip, knee; +, flexion/−, extension; ankle, +, dorsiflexion/−, plantar flexion.

The Pearson's correlation coefficients for the knee joint revealed strong associations with *r* = 0.96 (95% CI: 0.905–0.984) for front leg heel-off, *r* = 0.93 (95% CI: 0.829–0.970) for heel-strike, *r* = 0.99 (95% CI: 0.986–0.998) for rear leg heel-off, *r* = 0.98 (95% CI: 0.958–0.993) for heel strike, all indicating extremely large correlations. Similarly, at the ankle joint, significant correlations were found, with *r* = 0.83 (95% CI: 0.629–0.927) for front leg heel-off, *r* = 0.83 (95% CI: 0.634–0.928) for heel strike, *r* = 0.82 (95% CI: 0.610–0.923) for rear leg heel-off and *r* = 0.84 (95% CI: 0.658–0.934) heel strike, all indicating very large correlations. For the hip joints, the correlation coefficients were *r* = 0.71 (95% CI: 0.419–0.873) for front leg heel-off, *r* = 0.90 (95% CI: 0.774–0.959) for heel strike, and *r* = 0.81 (95% CI: 0.588–0.918) for rear leg heel-off, reflecting very large to extremely large correlations. However, the correlation for heel strike of the rear leg hip was not significant.

**Figure 4:**
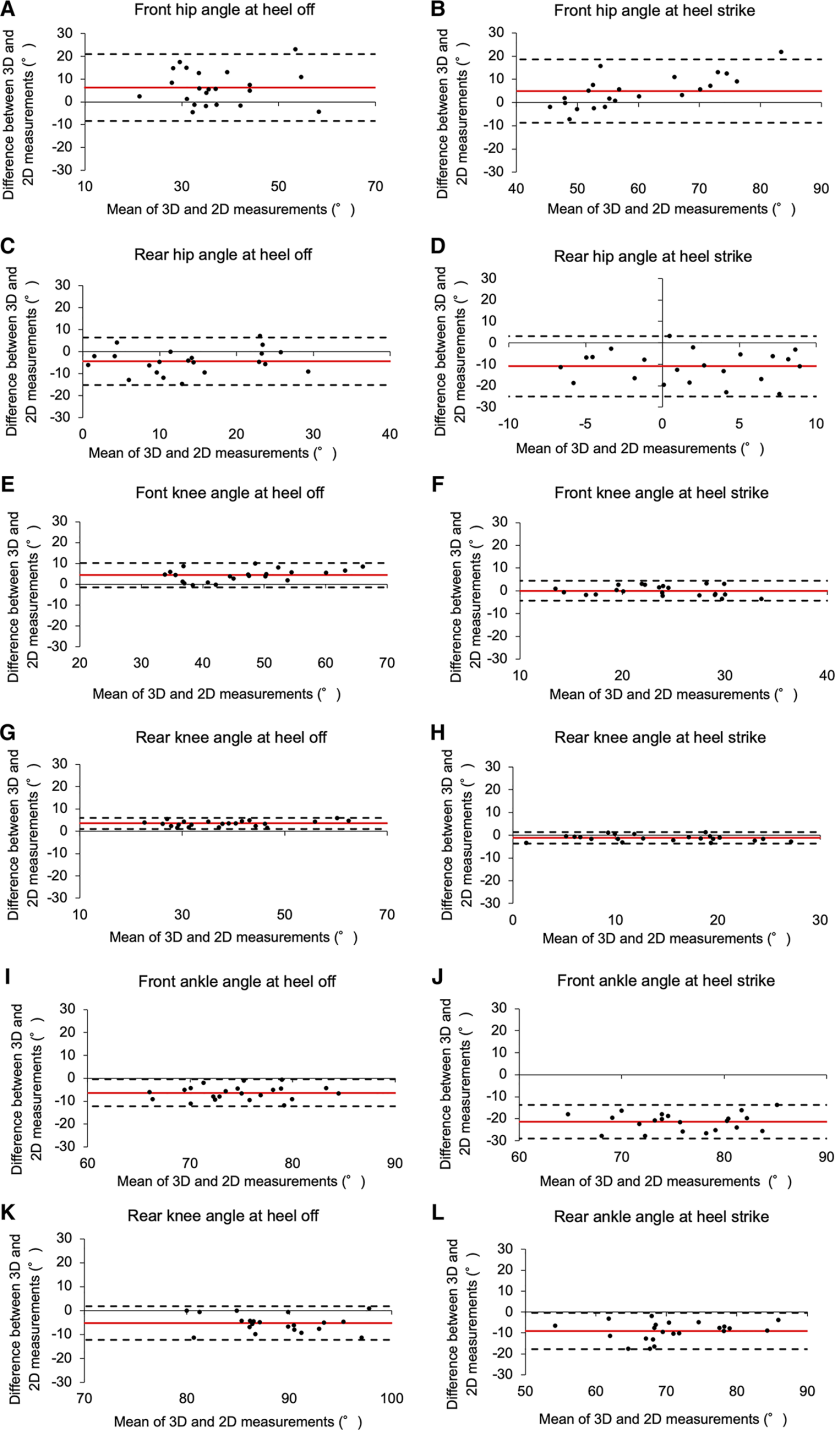
Bland-Altman plot comparing 3D motion analysis and 2D video analysis. (**A**) Front hip angle at heel off, (**B**) Front hip angle at heel strike, (**C**) Rear hip angle at heel off, (**D**) Rear hip angle at heel strike, (**E**) Font knee angle at heel off, (**F**) Front knee angle at heel strike, (**G**) Rear knee angle at heel off, (**H**) Rear knee angle at heel strike, (**I**) Front ankle angle at heel off, (**J**) Front ankle angle at heel strike, (**K**) Rear knee angle at heel off, (**L**) Rear ankle angle at heel strike.

In the Bland-Altman plot, the knee joint exhibited smaller mean differences, with 4.31° [limits of agreement (LOA) −1.46 to −10.08] for heel-off and 0.001° (LOA −4.35 to −4.34) for heel-strike in the front leg and 3.55° (LOA 1.07–6.03) for heel-off and −1.17° (LOA −3.76–1.42) for heel-strike in the rear leg, compared to the ankle and hip joints (Table 2, Figures 4E-H). At the ankle joint, the heel-off and heel-strike of the front leg were −6.35° (LOA −12.29 to −0.42) and −21.31° (LOA −28.98 to −13.64), respectively, whereas the heel-off and heel-strike of the rear leg were −5.23° (LOA −12.22–1.76) and −8.98° (LOA −17.64 to −0.31) respectively, all indicating an overestimation by 2D measurements (Table 2, Figures 4I-L). Particularly noteworthy was the heel strike of the front leg joint angle, exceeding 20° more than the angle measured by 3D. At the hip joint, the heel-off and heel-strike of the front leg were 6.28° (LOA −8.44–20.99) and 4.93° (LOA −8.74–18.61), respectively, and the heel-off and heel-strike of the rear leg were −4.33° (LOA −15.15–6.50) and −10.89° (LOA −24.98–3.20), respectively (Table 2, Figures 4A-D).

## 4 Discussion

In this study, we conducted a comparison between values derived from 2D video analysis and 3D motion analysis for lower limb joint angles for the fencing lunge motion, aiming to validate the effectiveness of 2D video analysis. The results revealed strong agreement in the front leg and rear leg knee joint angles at both heel-off and heel-strike, partially supporting our hypothesis. However, the hip and ankle joint angles of the front leg and rear leg did not yield highly valid data.

The knee joint angles of both the front and rear legs in this study align with the strongly correlated findings shown in previous studies that investigated lower limb joint angles using both 2D video analysis and 3D motion analysis methods (13, 7). In this study, the bias comparing 3D to 2D was within the range of bias reported in previous studies (1.5°–9.2°) at heel-off and heel-strike for both front and rear leg knee joints, indicating a high level of validity (13, 7). However, it is noteworthy that the heel strike of the front leg knee had a slightly lower correlation than the heel-off of the rear leg knee, the heel strike of the rear leg knee, and the heel-off of the front leg knee. This may arise from discrepancies between the angle of travel of the front foot and the direction of travel during the front foot heel strike. As confirmed in the study by Mousavi et al., 2020, overestimation or underestimation has been reported when the images captured by a 2D video camera deviate from the sagittal plane, such as in cases of internal rotation of the hip or ankle joint relative to the direction of travel (6). These factors were inferred to contribute to the slight diminished reliability in the results of this study as well.

The correlation between the ankle joint angles in the front and rear legs was very high (*r* = 0.82–0.84), yet the Bland and Altman plot revealed a bias ranging from −4.33° to −21.31° (Figure 4, I-L). This overestimation measurement bias is consistent with the findings in previous studies comparing 2D video analysis and 3D motion capture (13). The magnitude of the bias is influenced by the extent of the joint position shift on the y-axis (2D coordinate axis) within the measurement plane (sagittal plane) captured by a video camera (13). Furthermore, the Bland and Altman plot for the front foot ankle joint angle at the heel strike showed the largest bias at −21.31° (Table 3). This may be because joint positions were out of alignment in the x-axis as well as the y-axis on the sagittal plane captured by the digital video camera. In this study, a digital video camera was securely fixed vertically to the initial posture for recording. However, during the execution of the lunge, the distance from the initial posture position to the target (each participant's height × 1.5 times) was altered. The posture captured on the screen at the time of landing was considered to have resulted in an overestimation of the front ankle joint angle due to a gap created from the vertical angle set at the initial posture. These results indicate the importance of carefully considering the setting position of the digital video camera with respect to the joint positions on both the y- and x-axes in the sagittal plane. Future research is required to determine the optimal position and height from the ground for a digital video camera.

For the hip joints, the correlation of heel off in the front leg hip joint was very large, with *r* = 0.71; however, the correlation was not as high as that observed for the knee and ankle joints. For the rear leg hip joints, the correlation of heel strike was not significant, and the Bland Altman plot exhibited a notable bias of −10.89° (Table 2). A previous study comparing 2D and 3D measurements of hip angles in the sagittal plane during a single leg squat reported a strong correlation between the two measurements (7), and although the same 2D measurement method (the offset angle between the line drawn from the acromion marker to the greater trochanter marker and the line drawn from the greater trochanter marker to the lateral femoral epicondyle marker) was applied in this study, the agreement between the values in this study was low. One reason for this disparity could be the significant impact of fencing-specific movements on the 2D analysis method. Due to the characteristics of the fencing form, particularly in the front leg hip joint, the pelvis tilts forward toward the target during heel-off and heel strike. However, the trunk does not tilt forward more than the pelvis, resulting in an upright position of the upper body. The 2D analysis that calculates the hip angle using acromion markers may underestimate it compared to a 3D analysis that derives the angle based on the pelvis and femur. Moreover, most of the rear leg hip angles measured in the 2D images were overestimated. This result suggests that the characteristics of the upper body motion during the lunge may have influenced the measurements, particularly during heel-off and heel strike. During these phases, the acromion markers of the upper limbs were captured at an angle close to the coronal plane, resulting in a larger error compared to a single-leg squat as performed by Schurr et al. These results indicate that the line connecting the acromion and greater trochanter is likely not an appropriate means of calculating the hip angle in the sagittal plane in 2D measurements in the lunge, and future revalidation using different marker positions (e.g., a lower trunk marker instead of the acromion marker) or by validating different methods may help explore the possibility of 2D analysis. However, at this point, the use of 3D motion analysis is recommended to ensure the accuracy of the hip joint angle measurement.

This study holds certain limitations. First, the 2D analysis using a digital video camera positioned based on the initial posture might not fully capture the complex vertical and lateral movements inherent in the fencing lunge. This movement involves vertical and lateral movements because of the characteristics of the movement, taking a large step and striking with the sword; the distance traveled during landing is also involved. Therefore, the camera positions and lower limb joint angle calculation methods used in 2D video analysis are not always consistent with the joint angles captured on the screen (sagittal plane). Second, this is the first study to compare 3D motion analysis with 2D video analysis data in fencing, and the average of three trials was employed to identify trends in the overall data, whereas errors may have been offset by using the average as a representative value. Finally, this study only included skilled male fencers. Therefore, extending the research to female fencers and individuals across various age groups and competition levels is vital for broader applicability and understanding.

In this study, analysis was performed post-data transfer to a PC to improve the accuracy. However, considering its versatility in 2D, it is expected that in the future it will be validated in a framework that is easy to analyze using smartphone applications and other marker-less methods. Particularly, the knee joint angle, which was given excellent validity in this study, has been reported to be a performance factor contributing to fencers’ peak speed during lunge (2, 3). Further simplified measurement of the 2D analysis technique (6) is established, the use of immediate feedback during practice will become possible, and its use as a more convenient tool for instruction may be expanded. In addition, if we can comprehensively clarify the areas that can be measured in 2D video analysis by further examining the areas not investigated in this study (e.g., upper limbs), the findings could be more useful for coaching in competition settings.

## 5 Conclusion

This study aimed to validate 2D video analysis against 3D motion analysis by comparing lower limb joint angles (hip, knee, and ankle joints) during a fencing lunge. Notably, the front and rear leg knee joint angles demonstrated an extremely large correlation, suggesting the potential utility of 2D video analysis as an alternative to 3D motion analysis. However, for the ankle joint angle, the results revealed a substantial bias, emphasizing the need for further verification of the optimal video camera position and height in future studies. Regarding the hip joint angle, it was evident that the method used for calculating the angle in the 2D analysis was likely not appropriate, and 3D motion analysis is recommended as the method of choice at this time.

## Data Availability

The raw data supporting the conclusions of this article will be made available by the authors, without undue reservation.
